# Antimicrobial Resistance Patterns and Cumulative Antibiogram of Clinically Significant Bacteria From a Tertiary Hospital in Indonesia

**DOI:** 10.1155/ijm/1397226

**Published:** 2026-03-09

**Authors:** Rika Bur, Muhammad Alifian Remifta Putra, Nelly Puspandari, Syukrini Bahri, Evi Yuliana, Nadya Rashita Valentina Barus, Astari Arum Sari, Talitha Dhia Fairuza

**Affiliations:** ^1^ Department of Internal Medicine, Faculty of Medicine, Universitas YARSI, Jakarta, Indonesia; ^2^ YARSI Hospital, Jakarta, Indonesia; ^3^ Faculty of Medicine, Universitas Indonesia, Jakarta, Indonesia, ui.ac.id; ^4^ Institute of Epidemiology & Health Care, University College London, London, UK, ucl.ac.uk; ^5^ Faculty of Medicine, Universitas YARSI, Jakarta, Indonesia

**Keywords:** *Acinetobacter baumannii*, antibiogram, antimicrobial resistance, Indonesia, *Klebsiella pneumoniae*

## Abstract

**Background:**

Antimicrobial resistance (AMR) is a critical global health threat, with disproportionately high burdens in low‐ and middle‐income countries. In Indonesia, where antimicrobial stewardship initiatives are still developing, local antibiograms are scarce yet crucial to guide evidence‐based empirical therapy.

**Methods:**

We conducted a retrospective descriptive study of cumulative antibiogram data from YARSI Hospital, a tertiary care academic hospital in Jakarta, Indonesia, covering January–December 2024. Analysis followed Clinical and Laboratory Standards Institute (CLSI) M39‐A4 guidelines and was restricted to the first clinical isolate per species, patient, and specimen type. Susceptibility data were stratified by Gram classification, specimen source, and care setting, with semester‐wise comparisons (January–June vs. July–December).

**Results:**

Among 1782 clinical specimens, *Klebsiella pneumoniae* predominated among Gram‐negative isolates, exhibiting high multidrug resistance with ≤ 20% susceptibility to third‐generation cephalosporins, particularly in blood and ICU samples. *Acinetobacter baumannii*, the leading respiratory and ICU isolate, retained susceptibility only to amikacin (15%) and trimethoprim–sulfamethoxazole (25%). *Escherichia coli* from urine remained susceptible to ceftazidime (78%), cefepime (81%), meropenem (95%), and amikacin (93%). Gram‐positive isolates, including *Staphylococcus epidermidis* and *Enterococcus faecalis*, were largely susceptible to vancomycin and linezolid, although frequent coagulase‐negative staphylococcal bacteremia suggested challenges in aseptic technique.

**Conclusion:**

The persistence of multidrug‐resistant *K. pneumoniae* and *A. baumannii* highlights the urgent need for targeted antimicrobial stewardship in Indonesian tertiary hospitals. Routine, stratified antibiogram reporting is essential to guide empirical therapy, inform policy, and curb the progression of AMR.

## 1. Introduction

Antimicrobial resistance (AMR) is among the most pressing global health threats, undermining the effectiveness of antimicrobial agents and threatening decades of progress in infectious disease management [1–3]. The World Health Organization (WHO) has identified AMR as a top public health priority due to its significant contribution to preventable morbidity and mortality worldwide [2]. Recent estimates suggest that resistant bacterial infections were associated with nearly 5 million deaths in 2019, a figure projected to rise without coordinated containment strategies [3]. In healthcare settings, AMR leads to delayed recovery, prolonged hospitalization, increased healthcare costs, and severely limited therapeutic options, particularly for infections caused by multidrug‐resistant (MDR) and extensively drug‐resistant (XDR) organisms [4].

The burden of AMR is especially severe in low‐ and middle‐income countries (LMICs), where over‐the‐counter antibiotic access, limited diagnostic capacity, and inconsistent infection prevention and control practices accelerate the spread of resistant pathogens [5]. Indonesia exemplifies these challenges in which national surveillance reports document high rates of MDR organisms in tertiary care facilities, yet routine susceptibility data to guide empirical therapy remain scarce [6–9]. These gaps contribute to inappropriate prescribing practices and foster the persistence and spread of resistant strains, particularly in high‐risk clinical environments such as intensive care units (ICUs) [7, 9].

Cumulative hospital antibiograms are a cornerstone of antimicrobial stewardship programs. They summarize local susceptibility patterns, support evidence‐based empirical therapy, and help track emerging resistance trends [10]. When developed in line with standardized methodologies, such as those outlined by the Clinical and Laboratory Standards Institute (CLSI), antibiograms can inform institutional treatment guidelines and strengthen stewardship initiatives [11]. However, regular and stratified antibiogram reporting remains uncommon in many Indonesian hospitals, limiting the ability to tailor empirical treatment to local resistance ecology [6, 9].

This study reports the 2024 cumulative antibiogram of YARSI Hospital, a tertiary academic center in Jakarta, Indonesia. It consolidates susceptibility data from blood, urine, lower respiratory tract, and ICU‐derived isolates across two surveillance periods. To our knowledge, this is the first cumulative antibiogram report from a tertiary academic hospital in Jakarta that stratifies susceptibility by specimen type, care setting (ICU vs. non‐ICU), and semester within a single surveillance year, thereby complementing national AMR surveillance with actionable, institution‐specific data.

## 2. Methods

### 2.1. Study Setting, Design, and Period

A retrospective descriptive study was conducted at YARSI Hospital, a private tertiary academic hospital located in Central Jakarta, Indonesia, from January to December 2024. The hospital has a capacity of approximately 500 beds and provides inpatient and outpatient services across multiple specialties, including intensive care, surgery, internal medicine, pediatrics, and obstetrics and gynecology. The Clinical Microbiology Laboratory at YARSI Hospital routinely performs diagnostic cultures and antimicrobial susceptibility testing (AST) to support patient management and hospital‐wide infection control.

### 2.2. Study Population

All clinical specimens submitted to the Clinical Microbiology Laboratory for culture and AST during the study period were considered. Specimens originated from both inpatient and outpatient departments, including ICUs. To ensure data representativeness and avoid duplication, only the first isolate of a given bacterial species per patient per specimen type was included in the analysis, in accordance with CLSI M39‐A4 guidelines [9]. Records with incomplete microbiological data were excluded.

### 2.3. Data Collection

Microbiological records were retrieved retrospectively from the laboratory information system for the surveillance period. Extracted data included specimen type, bacterial species identified, and antimicrobial susceptibility profiles. Susceptibility data were stratified by Gram classification, specimen source (blood, urine, and lower respiratory tract), and patient care setting (ICU vs. non‐ICU). To assess temporal variation, isolates were also compared between two surveillance intervals: January–June 2024 (first semester) and July–December 2024 (second semester).

### 2.4. Laboratory Methods

All specimens were processed following standard bacteriological procedures. Identification and AST were performed using a combination of conventional microbiological methods and automated systems (VITEK 2, bioMérieux, France). Where applicable, initial culture media included blood agar, MacConkey agar, and chocolate agar (Oxoid, United Kingdom). Colonies were identified based on morphology, Gram stain, and confirmatory biochemical or automated system profiles.

AST was performed using the Kirby–Bauer disk diffusion method on Mueller–Hinton agar (Oxoid, United Kingdom) and/or automated broth microdilution via the VITEK 2 system, in accordance with CLSI 2024 guidelines. The bacterial suspension was standardized to 0.5 McFarland prior to inoculation. Plates were incubated at 35°C–37°C for 18–24 h.

The following antimicrobial agents were tested, with disk potencies where applicable:

• *β*‐Lactams: cefotaxime (30 *μ*g), ceftriaxone (30 *μ*g), ceftazidime (30 *μ*g), and cefepime (30 *μ*g)
• *β*‐Lactam/*β*‐lactamase inhibitors: ampicillin–sulbactam (10/10 *μ*g) and amoxicillin–clavulanate (20/10 *μ*g)
• Carbapenems: imipenem (10 *μ*g) and meropenem (10 *μ*g)
• Aminoglycosides: gentamicin (10 *μ*g) and amikacin (30 *μ*g)
• Fluoroquinolones: ciprofloxacin (5 *μ*g) and levofloxacin (5 *μ*g)
• Others: trimethoprim–sulfamethoxazole (1.25/23.75 *μ*g), clindamycin (2 *μ*g), vancomycin (MIC testing only), linezolid (10 *μ*g), and fosfomycin (200 *μ*g; where available)

### 2.5. Quality Control

Quality control was performed in accordance with CLSI recommendations. Reference strains used included *Escherichia coli* ATCC 25922, *Staphylococcus aureus* ATCC 25923, *Pseudomonas aeruginosa* ATCC 27853, and *Enterococcus faecalis* ATCC 29212. Media sterility was routinely checked by incubating 5% of each batch overnight, while performance testing was conducted using QC strains prior to culture and AST. Antibiotic disks were sourced from Oxoid (United Kingdom), and the selection of agents was based on CLSI guidelines and local availability.

### 2.6. Data Analysis and Interpretation

Data were entered and analyzed using Microsoft Excel 2021. Descriptive statistics were used to summarize the frequencies of organisms and the proportions of antimicrobial susceptibility. In addition, inferential analyses were conducted to evaluate semester‐wise differences in surveillance distributions. Differences in the distribution of specimen types, sample origins, and organism groups between Semester I (January–June) and Semester II (July–December) were assessed using Pearson’s chi‐square tests on aggregated counts.

To satisfy statistical assumptions and reduce sparse cells, low‐frequency categories were collapsed into clinically meaningful groups prior to inferential testing. Where overall chi‐square tests were significant, post hoc analyses were limited to prespecified, high‐volume categories with direct clinical relevance to minimize multiple testing and avoid overinterpretation of surveillance data. Post hoc comparisons used 2 × 2 chi‐square tests with Fisher’s exact tests where appropriate.

## 3. Results

### 3.1. Study Characteristics

During the 2024 surveillance period, a total of 1782 clinical specimens were processed at the YARSI Hospital Clinical Microbiology Laboratory. The majority of samples were obtained from hospitalized patients, particularly from blood, urine, and respiratory tract specimens. Data collection was stratified into two semesters: January–June (Semester I) and July–December (Semester II).

#### 3.1.1. Sample Types

**Figure 1:**
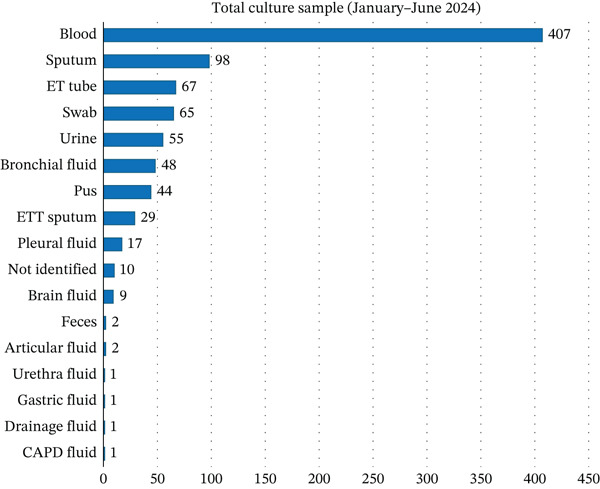
Total culture sample from the first semester (January–June 2024).

**Figure 2:**
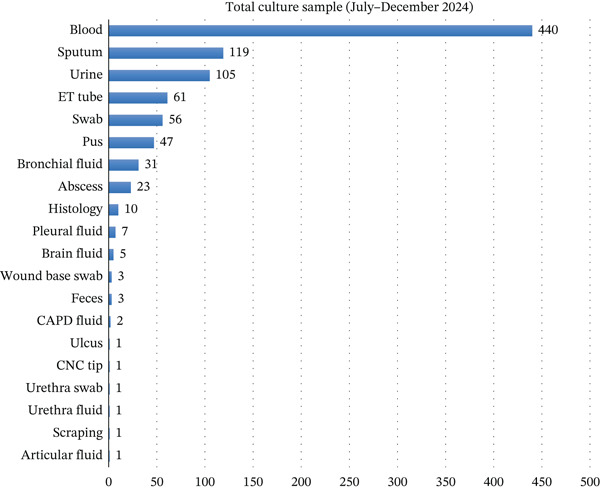
Total culture sample from the second semester (July–December 2024).

Blood was the most frequently submitted specimen in both semesters, comprising 407 cultures in Semester I, as shown in Figure 1, and 440 cultures in Semester II, as shown in Figure 2. Lower respiratory tract specimens (sputum, endotracheal tube [ETT] secretions, and bronchial washings) also contributed substantially, reflecting a clinical focus on respiratory infections. Urine, pus, and wound swabs were additional common specimen types.

The distribution of specimen types differed significantly between Semester I and Semester II (Pearson’s *χ* ^2^(7) = 23.07, *p* = 0.002; aggregated *N* = 1775). In post hoc analysis, urine specimens accounted for a significantly higher proportion of samples in Semester II compared with Semester I (*χ* ^2^(1) = 13.62, *p* < 0.001; Fisher’s exact *p* < 0.001).

#### 3.1.2. Clinical Locations

**Figure 3:**
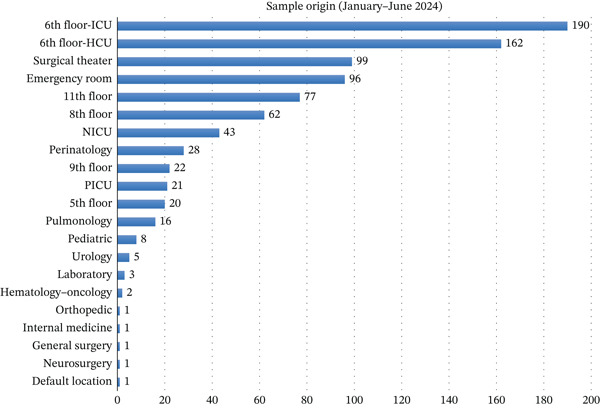
Sample origin from the first semester (January–June 2024).

**Figure 4:**
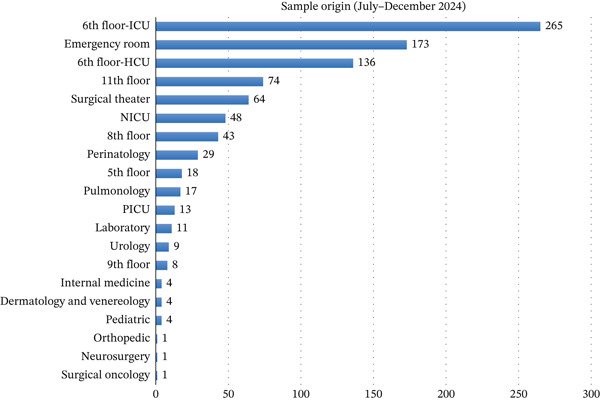
Sample origin from the second semester (July–December 2024).

Most specimens originated from high‐acuity settings, as summarized by Figures 3 and 4. The ICU (sixth‐floor ICU) consistently accounted for the largest proportion (190 specimens in Semester I and 265 in Semester II), followed by the emergency department, high care units (HCUs), and operating rooms. Other notable contributors included the NICU, pediatric wards, and pulmonology clinics.

After collapsing clinical locations into major service categories, the distribution of sample origins differed significantly between semesters (*χ* ^2^(7) = 37.42, *p* < 0.001; aggregated *N* = 1777). In post hoc analyses, the proportion of specimens originating from the emergency department increased significantly in Semester II (*χ* ^2^(1) = 20.14, *p* < 0.001; Fisher’s exact *p* < 0.001). In contrast, the proportion of specimens originating from the ICU/HCU did not differ significantly between semesters (*χ* ^2^(1) = 1.23, *p* = 0.267; Fisher’s exact *p* = 0.269).

#### 3.1.3. Microbial Isolates

**Figure 5:**
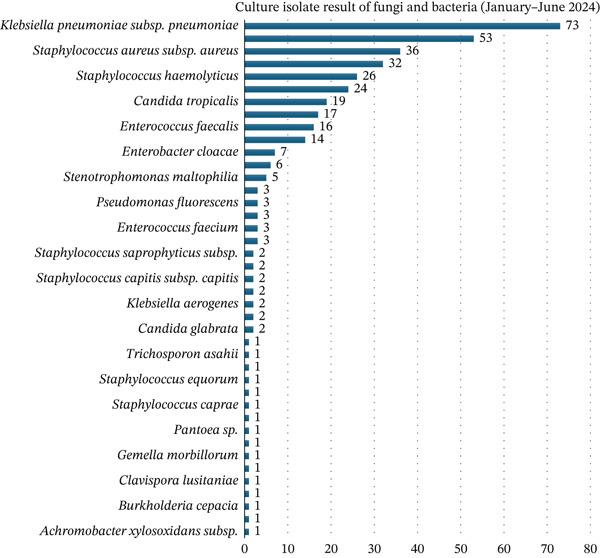
Culture isolate result from the first semester (January–June 2024).

**Figure 6:**
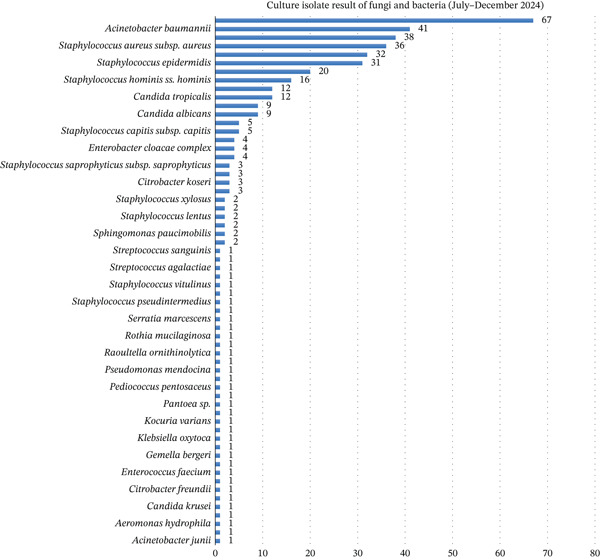
Culture isolate result from the second semester (July–December 2024).

Across both semesters, *Klebsiella pneumoniae* and *Acinetobacter baumannii* were the most frequently isolated Gram‐negative organisms, as shown in Figures 5 and 6. In Semester I, *K. pneumoniae* accounted for 73 isolates, while in Semester II, *A. baumannii* predominated with 67 isolates. Among Gram‐positive organisms, *S. aureus*, *Staphylococcus epidermidis*, and other coagulase‐negative staphylococci (CoNS) (*Staphylococcus hominis* and *Staphylococcus haemolyticus*) were most common. Opportunistic fungi, including *Candida tropicalis* and *Candida albicans*, were also observed. The isolate distribution reflected the clinical burden of hospital‐acquired infections, particularly in critical care units.

When isolates were grouped into major organism categories, the distribution of organisms differed significantly between Semester I and Semester II (Pearson’s *χ* ^2^(6) = 115.96, *p* < 0.001; *N* = 453). This distributional shift was primarily driven by an increased proportion of nonfermenting Gram‐negative bacilli in Semester II compared with Semester I (31.7% vs. 4.7%; *χ* ^2^(1) = 55.91, *p* < 0.001; Fisher’s exact *p* < 0.001).

### 3.2. Overview of Clinical Isolates

Of the 1782 processed specimens, *K. pneumoniae* was the most frequent Gram‐negative isolate (19.6%), while *S. epidermidis* was the most common Gram‐positive organism in blood cultures. All analyses were deduplicated per CLSI M39‐A4 guidelines, with only the first isolate per species per patient per specimen type included. Organism–antibiotic combinations with < 30 isolates were excluded from antibiogram reporting to ensure statistical validity. Antibiogram interpretation focused on blood, urine, and lower respiratory tract isolates. Where provided, the number of isolates tested per antibiotic is indicated in parentheses; if no number is listed, susceptibility testing was conducted on all isolates of that species. Color coding was used in antibiogram tables to indicate sensitivity thresholds: orange for < 50% susceptible, yellow for 50%–75%, and green for ≥ 76%.

### 3.3. Gram‐Negative Bacteria

Among Gram‐negative bacilli, *E. coli*, *K. pneumoniae*, *P. aeruginosa*, and *A. baumannii* were the predominant pathogens. *E. coli* showed high resistance to ampicillin but retained moderate‐to‐high susceptibility to aminoglycosides, fluoroquinolones, and carbapenems. *K. pneumoniae* exhibited broad resistance across multiple antibiotic classes, including third‐generation cephalosporins, *β*‐lactam/*β*‐lactamase inhibitor combinations, and fluoroquinolones, raising concern for ESBL and carbapenemase activity. *P. aeruginosa* maintained moderate susceptibility to ceftazidime, cefepime, and aminoglycosides. *A. baumannii* was the most resistant organism, showing extensive drug resistance with limited susceptibility primarily to amikacin and trimethoprim–sulfamethoxazole. Some antibiotic–organism combinations lacked data due to limited isolate numbers or testing constraints.

#### 3.3.1. Blood Isolates

*K. pneumoniae* and *P. aeruginosa* were the dominant Gram‐negative pathogens in blood cultures. *K. pneumoniae* isolates exhibited poor susceptibility (< 50%) to most cephalosporins and penicillin combinations, although 65.2% remained susceptible to meropenem and 56.5% to amikacin in Semester I (Tables 1 and 2). *P. aeruginosa* retained moderate susceptibility to ceftazidime and aminoglycosides (Tables 1 and 2).

**Table 1:** Antibiotic susceptibility (%) of Gram‐negative bacilli from blood specimens to antibiotics (first semester, 2024).

Bacteria	*N*	CTX	CTR	CAZ	FEP	AMC	SAM	IPM	MEM	GEN	AMK	CIP	LVX	SXT
*Klebsiella pneumoniae* subsp. *pneumoniae*	23		4.30	21.70	39.10		4.30		65.20	21.70	56.50	1.40		65.20
*Pseudomonas aeruginosa*	5			40	40				40		60	40		
*Acinetobacter baumannii*	4		25	25	25		25		25	75	75	25		50
*Escherichia coli*	3		33.70	66.70	100		100		100	66.70	100	33.30		66.70
*Enterobacter cloacae*	2		0	0	0		0		100	0	50	0		50
Other Gram‐negative bacteria	2		100 (1)	100 (1)	100 (1)		0 (1)		100 (1)	100 (1)	100 (1)	100 (1)		100

Abbreviations: AMC, amoxicillin–clavulanate; AMK, amikacin; CAZ, ceftazidime; CIP, ciprofloxacin; CTR, ceftriaxone; CTX, cefotaxime; FEP, cefepime; GEN, gentamicin; IPM, imipenem; LVX, levofloxacin; MEM, meropenem; SAM, ampicillin–sulbactam; SXT, trimethoprim–sulfamethoxazole.

**Table 2:** Antibiotic susceptibility (%) of Gram‐negative bacilli from blood specimens to antibiotics (second semester, 2024).

Bacteria	*N*	CTX	CTR	CAZ	FEP	AMC	SAM	IPM	MEM	GEN	AMK	CIP	LVX	SXT
*Klebsiella pneumoniae* subsp. *pneumoniae*	24		41.70	16.70	58.30		0		66.70	12.50	66.70	83.30		66.70
*Acinetobacter baumannii*	6		0	16.70	16.70		16.70		16.70	16.70	33.30	16.70		33.30
*Escherichia coli*	6		16.70	83.30	83.30		33.30		83.30	50	100	50		50
*Pseudomonas aeruginosa*	3			100	100				100		100	100		
Other Gram‐negative bacteria	5		20	20	50 (4)		40		80	40	60	25 (4)		0

Abbreviations: AMC, amoxicillin–clavulanate; AMK, amikacin; CAZ, ceftazidime; CIP, ciprofloxacin; CTR, ceftriaxone; CTX, cefotaxime; FEP, cefepime; GEN, gentamicin; IPM, imipenem; LVX, levofloxacin; MEM, meropenem; SAM, ampicillin–sulbactam; SXT, trimethoprim–sulfamethoxazole.

#### 3.3.2. Urine Isolates

*E. coli* was the leading uropathogen and retained high susceptibility to ceftazidime, cefepime, meropenem, and amikacin (> 88%, shown in Tables 3 and 4). Resistance was more pronounced to ampicillin–sulbactam and ciprofloxacin. In contrast, *K. pneumoniae* isolates from urine displayed markedly lower susceptibility across all tested agents (Tables 3 and 4).

**Table 3:** Antibiotic susceptibility (%) of Gram‐negative bacilli from urine specimens to antibiotics (first semester, 2024).

Bacteria	*N*	CTX	CTR	CAZ	FEP	AMC	SAM	IPM	MEM	GEN	AMK	CIP	LVX	SXT	FOS
*Escherichia coli*	9		22.20	88.90	88.90		44.40		100	33.30	100	44.40		55.60	
*Klebsiella pneumoniae* subsp. *pneumoniae*	4		0	0	50		0		100	0	74	0		0	

Abbreviations: AMC, amoxicillin–clavulanate; AMK, amikacin; CAZ, ceftazidime; CIP, ciprofloxacin; CTR, ceftriaxone; CTX, cefotaxime; FEP, cefepime; GEN, gentamicin; IPM, imipenem; LVX, levofloxacin; MEM, meropenem; SAM, ampicillin–sulbactam; SXT, trimethoprim–sulfamethoxazole.

**Table 4:** Antibiotic susceptibility (%) of Gram‐negative bacilli from urine specimens to antibiotics (second semester, 2024).

Bacteria	*N*	CTX	CTR	CAZ	FEP	AMC	SAM	IPM	MEM	GEN	AMK	CIP	LVX	SXT	FOS
*Escherichia coli*	14		21.40	64.30	75		35.70		92.90	64.30	92.90	42.90		64.30	
*Klebsiella pneumoniae* subsp. *pneumoniae*	4		0	0	0		0		75	0	25	0		50	
*Pseudomonas aeruginosa*	3			66.70	66.70				66.7		33.30	66.70			
*Proteus mirabilis*	2		50	100	100		100		100	50	100	50		50	
Other Gram‐negative bacteria	4		33.30 (3)	33.30 (3)	33.30 (3)		0 (1)		33.30 (3)	33.30 (3)	33.30 (3)	33.30 (3)		50	

Abbreviations: AMC, amoxicillin–clavulanate; AMK, amikacin; CAZ, ceftazidime; CIP, ciprofloxacin; CTR, ceftriaxone; CTX, cefotaxime; FEP, cefepime; GEN, gentamicin; IPM, imipenem; LVX, levofloxacin; MEM, meropenem; SAM, ampicillin–sulbactam; SXT, trimethoprim–sulfamethoxazole.

#### 3.3.3. Lower Respiratory Tract Isolates

Respiratory tract specimens were dominated by *A. baumannii* and *K. pneumoniae*. *A. baumannii* exhibited broad resistance but retained activity to amikacin (78.8%) and SXT (57.6%). *K. pneumoniae* from respiratory sources showed poor susceptibility, with < 32% sensitivity to most agents except amikacin (Tables 5 and 6).

**Table 5:** Antibiotic susceptibility (%) of Gram‐negative bacilli from sputum of lower respiratory tract specimens to antibiotics (first semester, 2024).

Bacteria	*N*	CTX	CTR	CAZ	FEP	AMC	SAM	IPM	MEM	GEN	AMK	CIP	LVX	SXT
*Acinetobacter baumannii*	33		0	6.10	6.10		6.10		6.10	12.10	78.80	6.10		57.60
*Klebsiella pneumoniae* subsp. *pneumoniae*	22		0	4.50	0 (21)		13.60		0	31.80	50	27.30		63.60
*Pseudomonas aeruginosa*	13			38.50	30.80				30.80		38.50	38.50		
*Escherichia coli*	8		0	25	75		25		87.50	25	100	12.50		50
*Enterobacter cloacae*	3		0	0			0		100	66.70	100	0		0
Other Gram‐negative bacteria	8		0 (4)	25 (4)	100 (3)		0 (3)		100 (4)	75 (4)	75 (4)	50 (4)		57.10 (7)

Abbreviations: AMC, amoxicillin–clavulanate; AMK, amikacin; CAZ, ceftazidime; CIP, ciprofloxacin; CTR, ceftriaxone; CTX, cefotaxime; FEP, cefepime; GEN, gentamicin; IPM, imipenem; LVX, levofloxacin; MEM, meropenem; SAM, ampicillin–sulbactam; SXT, trimethoprim–sulfamethoxazole.

**Table 6:** Antibiotic susceptibility (%) of Gram‐negative bacilli from sputum of lower respiratory tract specimens to antibiotics (second semester, 2024).

Bacteria	*N*	CTX	CTR	CAZ	FEP	AMC	SAM	IPM	MEM	GEN	AMK	CIP	LVX	SXT
*Acinetobacter baumannii*	19		0	22.2 (18)	21.10		26.30		33.30 (18)	21.10	94.70	22.20 (18)		78.90
*Klebsiella pneumoniae* subsp. *pneumoniae*	13		7.70	15.40	25 (12)		15.40		38.50	23.10	30.80	7.70		84.60
*Pseudomonas aeruginosa*	10			20	30				20		20	20		
*Escherichia coli*	5		0	40	75 (4)		20		100	80	100	40		60
*Enterobacter cloacae* complex	2		100	100	100		0		100	100	100	100		100
Other Gram‐negative bacteria	7		40 (5)	40 (5)	60 (5)		33.30 (3)		80 (5)	40 (5)	80 (5)	40 (5)		42.90

Abbreviations: AMC, amoxicillin–clavulanate; AMK, amikacin; CAZ, ceftazidime; CIP, ciprofloxacin; CTR, ceftriaxone; CTX, cefotaxime; FEP, cefepime; GEN, gentamicin; IPM, imipenem; LVX, levofloxacin; MEM, meropenem; SAM, ampicillin–sulbactam; SXT, trimethoprim–sulfamethoxazole.

#### 3.3.4. ICU‐Specific Gram‐Negative Isolates

ICU‐derived Gram‐negative isolates were primarily *A. baumannii*, exhibiting MDR phenotypes with susceptibility only to amikacin (71%) and SXT (51.6%). *K. pneumoniae* from ICU patients showed moderate susceptibility to cefepime (35%) and SXT (75%) but significant resistance to carbapenems and fluoroquinolones (Tables 7 and 8).

**Table 7:** Antibiotic susceptibility (%) of Gram‐negative bacilli from ICU specimens to antibiotics (first semester, 2024).

Bacteria	*N*	CTX	CTR	CAZ	FEP	AMC	SAM	IPM	MEM	GEN	AMK	CIP	LVX	SXT
*Acinetobacter baumannii*	31		0	0	3.20		6.50		0	6.50	71	3.20		51.60
*Klebsiella pneumoniae* subsp. *pneumoniae*	20		5	15	35		15		45	35	45	25		75
*Pseudomonas aeruginosa*	8			50	50				50		50	50		
*Escherichia coli*	4		0	25	25		0		75	25	100	25		50
*Enterobacter cloacae*	2		0	0	0 (1)		0		100	0	50	0		50
Other Gram‐negative bacteria	5		0 (1)	0 (1)	0 (1)				0 (1)	0 (1)	0 (1)	0 (2)		0 (3)

Abbreviations: AMC, amoxicillin–clavulanate; AMK, amikacin; AMP, ampicillin; CAZ, ceftazidime; CIP, ciprofloxacin; CTR, ceftriaxone; CTX, cefotaxime; FEP, cefepime; GEN, gentamicin; IPM, imipenem; LVX, levofloxacin; MEM, meropenem; SAM, ampicillin–sulbactam; SXT, trimethoprim–sulfamethoxazole.

**Table 8:** Antibiotic susceptibility (%) of Gram‐negative bacilli from ICU specimens to antibiotics (second semester, 2024).

Bacteria	*N*	CTX	CTR	CAZ	FEP	AMC	SAM	IPM	MEM	GEN	AMK	CIP	LVX	SXT
*Acinetobacter baumannii*	30		0	17.20 (29)	16.70		20		24.10 (29)	16.70	76.70	13.80 (29)		63.30
*Klebsiella pneumoniae* subsp. *pneumoniae*	28		0	14.30	32.10		14.30		28.60	14.30	32.10	7.10		78.60
*Pseudomonas aeruginosa*	17			41.20	41.20				41.20		35.30	41.20		
*Escherichia coli*	5		0	80	80		20		100	40	100	20		20
*Citrobacter koseri*	2		100	100	100		50		100	100	100	100		100
Other Gram‐negative bacteria	7		0 (5)	0 (5)	40 (5)		33.30 (3)		60 (5)	40 (5)	80 (5)	20 (5)		57.10

Abbreviations: AMC, amoxicillin–clavulanate; AMK, amikacin; CAZ, ceftazidime; CIP, ciprofloxacin; CTR, ceftriaxone; CTX, cefotaxime; FEP, cefepime; GEN, gentamicin; IPM, imipenem; LVX, levofloxacin; MEM, meropenem; SAM, ampicillin–sulbactam; SXT, trimethoprim–sulfamethoxazole.

### 3.4. Gram‐Positive Bacteria

Among Gram‐positive cocci, *S. aureus*, *S. hominis*, *S. haemolyticus*, and *E. faecalis* were commonly identified. Most isolates remained highly susceptible to vancomycin and linezolid, indicating preserved effectiveness of glycopeptides and oxazolidinones. *S. aureus* also showed good susceptibility to clindamycin and moderate susceptibility to fluoroquinolones. *E. faecalis* retained sensitivity to ampicillin and vancomycin but exhibited resistance to erythromycin. CoNS displayed reduced susceptibility to penicillin and macrolides, though they remained susceptible to vancomycin and linezolid. Incomplete data for some organism–antibiotic combinations were due to limited testing or low isolate counts.

#### 3.4.1. Blood Isolates

CoNS (mainly *S. hominis* and *S. epidermidis*) dominated, with uniform susceptibility to VAN and LNZ but high resistance to *β*‐lactams and macrolides. *E. faecalis* remained susceptible to ampicillin and VAN (Tables 9 and 10).

**Table 9:** Antibiotic susceptibility (%) of Gram‐positive cocci from blood specimens to antibiotics (first semester, 2024).

Bacteria	*N*	PEN	AMP	GEN	ERY	CIP	LVX	CLI	SXT	VAN	LNZ
*Staphylococcus hominis* subsp. *hominis* (CoNS)	13	0		100	23.10	61.50	61.50		69.20	100	100
*Enterococcus faecalis*	12	100	100		0	50	50			100	100
*Staphylococcus haemolyticus* (CoNS)	12	0		25	16.70	25	25		75	100	100
*Staphylococcus aureus* subsp. *aureus*	11	18.20		63.60	90.90	45.50	54.50		90.90	100	100
*Staphylococcus epidermidis* (CoNS)	11	0 (10)		60 (10)	30 (10)	30 (10)	30 (10)		30 (10)	100 (10)	100 (10)
Other Gram‐positive bacteria	12	0 (6)	50 (2)	100 (4)	50 (6)	50 (6)	50 (6)		100 (4)	100 (6)	100 (6)

Abbreviations: AMP, ampicillin; CIP, ciprofloxacin; CLI, clindamycin; ERY, erythromycin; GEN, gentamicin; LNZ, linezolid; LVX, levofloxacin; PEN, penicillin; SXT, trimethoprim–sulfamethoxazole; VAN, vancomycin.

**Table 10:** Antibiotic susceptibility (%) of Gram‐positive cocci from blood specimens to antibiotics (second semester, 2024).

Bacteria	*N*	PEN	AMP	GEN	ERY	CIP	LVX	CLI	SXT	VAN	LNZ
*Staphylococcus epidermidis*	22	4.50		22.70	18.20	18.20	18.20		27.30	100	100
*Staphylococcus aureus* subsp. *aureus*	14	14.30		85.70	85.70	78.60	78.60		92.90	100	100
*Staphylococcus hominis* subsp. *hominis*	14	7.10		78.60	42.90	35.70	35.70		64.30	100	100
*Staphylococcus haemolyticus*	13	0		23.10	15.40	15.40	15.40		84.60	100	100
*Staphylococcus capitis* subsp. *capitis*	5	0		40	40	20	20		100	100	100
Other Gram‐positive bacteria	14	55.60 (9)		50 (6)	22.20 (9)	33.30 (9)	33.30 (9)		83.30 (6)	100 (9)	100 (9)

Abbreviations: AMP, ampicillin; CIP, ciprofloxacin; CLI, clindamycin; ERY, erythromycin; GEN, gentamicin; LNZ, linezolid; LVX, levofloxacin; PEN, penicillin; SXT, trimethoprim–sulfamethoxazole; VAN, vancomycin.

#### 3.4.2. Urine and Respiratory Isolates

*E. faecalis* and *S. haemolyticus* predominated in urine, showing resistance to fluoroquinolones but retaining susceptibility to VAN. Respiratory specimens yielded *S. aureus* and *S. epidermidis*, both demonstrating strong susceptibility to VAN, LNZ, and CLI (Tables 11 and 12).

**Table 11:** Antibiotic susceptibility (%) of Gram‐positive cocci from urine specimens to antibiotics (first semester, 2024).

Bacteria	*N*	PEN	AMP	GEN	ERY	CIP	LVX	CLI	SXT	VAN	LNZ
*Staphylococcus haemolyticus*	4	0		50	0	50	50		25	100	100
*Enterococcus faecalis*	3	100	100		0	0	0			100	100
Other Gram‐positive bacteria	3	33.30	100 (1)	100 (2)	33.30	0	0		50 (2)	100	100

Abbreviations: AMP, ampicillin; CIP, ciprofloxacin; CLI, clindamycin; ERY, erythromycin; GEN, gentamicin; LNZ, linezolid; LVX, levofloxacin; PEN, penicillin; SXT, trimethoprim–sulfamethoxazole; VAN, vancomycin.

**Table 12:** Antibiotic susceptibility (%) of Gram‐positive cocci from urine specimens to antibiotics (second semester, 2024).

Bacteria	*N*	PEN	AMP	GEN	ERY	CIP	LVX	CLI	SXT	VAN	LNZ
*Enterococcus faecalis*	5	60	60		0	40	40			100	100
*Staphylococcus haemolyticus*	2	0		50	50	50	50		50	100	100
Other Gram‐positive bacteria	3	66.70	100 (1)	100 (2)	66.70	66.70	66.70		100 (2)	100	100

Abbreviations: AMP, ampicillin; CIP, ciprofloxacin; CLI, clindamycin; ERY, erythromycin; GEN, gentamicin; LNZ, linezolid; LVX, levofloxacin; PEN, penicillin; SXT, trimethoprim–sulfamethoxazole; VAN, vancomycin.

#### 3.4.3. ICU‐Specific Gram‐Positive Isolates

In the ICU, *S. haemolyticus* and *S. epidermidis* dominated Gram‐positive isolates. These showed persistent resistance to *β*‐lactams and fluoroquinolones but were uniformly susceptible to VAN and LNZ (Tables 13 and 14). The high frequency of CoNS from blood cultures raises concerns regarding contamination or suboptimal aseptic sampling.

**Table 13:** Antibiotic susceptibility (%) of Gram‐positive cocci from ICU specimens to antibiotics (first semester, 2024).

Bacteria	*N*	PEN	AMP	GEN	ERY	CIP	LVX	CLI	SXT	VAN	LNZ
	5	0 (4)		0 (4)	0 (4)	0 (4)	0 (4)		75 (4)	100 (4)	100 (4)
*Staphylococcus epidermidis*	4	0		25	0	0	0		0	100	100
*Enterococcus faecalis*	3	100	100		0	33.30	33.30			100	100
*Staphylococcus aureus* subsp. *aureus*	3	33.30		66.70		66.70	66.70		100	100	100
*Enterococcus faecium*	2	50	50		0	0	0			100	100
Other Gram‐positive bacteria	4	0 (2)		100 (2)	50 (2)	100 (2)	100 (2)		100 (2)	100 (2)	100 (2)

Abbreviations: AMP, ampicillin; CIP, ciprofloxacin; CLI, clindamycin; ERY, erythromycin; GEN, gentamicin; LNZ, linezolid; LVX, levofloxacin; PEN, penicillin; SXT, trimethoprim–sulfamethoxazole; VAN, vancomycin.

**Table 14:** Antibiotic susceptibility (%) of Gram‐positive cocci from ICU specimens to antibiotics (second semester, 2024).

Bacteria	*N*	PEN	AMP	GEN	ERY	CIP	LVX	CLI	SXT	VAN	LNZ
*Staphylococcus epidermidis*	14	0		14.30	7.10	7.10	7.10		7.10	100	100
*Staphylococcus hominis* subsp. *hominis*	5	0		80	0	0	0		20	100	100
*Staphylococcus aureus* subsp. *aureus*	4	0		100	100	100	100		100	100	100
*Staphylococcus haemolyticus*	4	0		0	0	0	0		75	100	100
*Kocuria kristinae*	2										
Other Gram‐positive bacteria	10	33.30 (6)	100 (1)	40 (5)	33.30 (6)	33.30 (6)	33.30 (6)		100 (5)	100 (6)	100 (6)

Abbreviations: AMP, ampicillin; CIP, ciprofloxacin; CLI, clindamycin; ERY, erythromycin; GEN, gentamicin; LNZ, linezolid; LVX, levofloxacin; PEN, penicillin; SXT, trimethoprim–sulfamethoxazole; VAN, vancomycin.

### 3.5. Temporal Comparison Between Semesters

Semester‐based analysis revealed minimal fluctuations in susceptibility trends for most organism–antibiotic combinations. However, a subtle decline in carbapenem susceptibility was observed among ICU‐derived *K. pneumoniae* isolates from the first semester to the second semester. *E. coli* susceptibility remained stable, while *A. baumannii* showed progressive reduction in sensitivity to previously active agents, such as amikacin and SXT, in the second semester.

**Figure 7:**
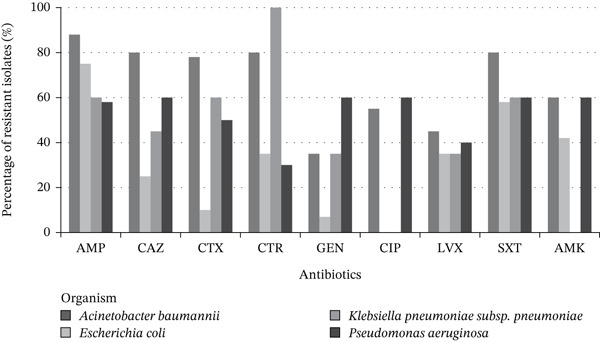
Antibiotic resistance trends: Gram‐negative organisms. The vertical axis shows the percentage of resistant isolates; the horizontal axis lists individual antibiotics. Each bar color represents a specific organism, as indicated in the legend. Abbreviations: AMC, amoxicillin–clavulanate; AMK, amikacin; AMP, ampicillin; CAZ, ceftazidime; CIP, ciprofloxacin; CTR, ceftriaxone; CTX, cefotaxime; FEP, cefepime; GEN, gentamicin; IPM, imipenem; LVX, levofloxacin; MEM, meropenem; SAM, ampicillin–sulbactam; SXT, trimethoprim–sulfamethoxazole.

Figure 7 illustrates the antibiotic resistance trends among major Gram‐negative organisms isolated in 2024, including *E. coli*, *K. pneumoniae*, *P. aeruginosa*, and *A. baumannii*. The bar chart shows the average percentage of resistance to a selection of commonly used antibiotics. Overall, *A. baumannii* demonstrated the highest levels of resistance across nearly all agents tested, consistent with an MDR phenotype. *K. pneumoniae* also exhibited elevated resistance, particularly to third‐generation cephalosporins and fluoroquinolones. In contrast, *E. coli* showed moderate resistance patterns, while *P. aeruginosa* displayed variable resistance, with some susceptibility retained to ceftazidime and gentamicin.

**Figure 8:**
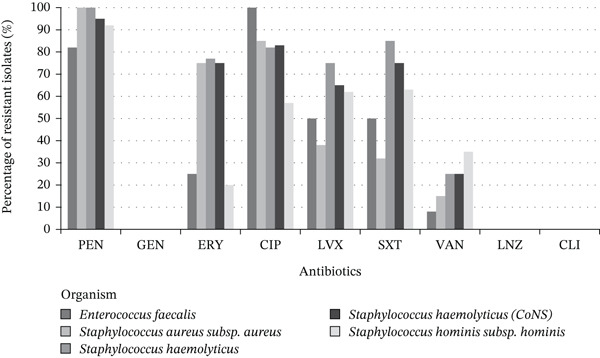
Antibiotic resistance trends: Gram‐positive organisms. The vertical axis shows the percentage of resistant isolates; the horizontal axis lists individual antibiotics. Each bar color represents a specific organism, as indicated in the legend. Abbreviations: AMP, ampicillin; CIP, ciprofloxacin; CLI, clindamycin; ERY, erythromycin; GEN, gentamicin; LNZ, linezolid; LVX, levofloxacin; PEN, penicillin; SXT, trimethoprim–sulfamethoxazole; VAN, vancomycin.

Figure 8 presents the resistance profiles of key Gram‐positive organisms, including *S. aureus*, *S. hominis*, *S. haemolyticus*, and *E. faecalis*. Resistance to penicillin and erythromycin was commonly observed, especially among CoNS. However, high susceptibility was generally preserved for vancomycin and linezolid across most species. These visual comparisons provide a clear overview of resistance burdens and support targeted empirical treatment decisions based on organism type and likely resistance mechanisms.

## 4. Discussion

### 4.1. AMR Landscape at YARSI Hospital

The 2024 cumulative antibiogram from YARSI Hospital in Jakarta demonstrates a substantial burden of MDR pathogens, particularly Gram‐negative bacilli isolated from ICU and respiratory specimens. By combining detailed stratification by ward, specimen source, and pathogen with strict adherence to current CLSI interpretive standards, this antibiogram provides a more methodologically rigorous and standardized depiction of local AMR than many previous Indonesian hospital reports, which have often focused on a single clinical unit or a limited group of organisms.

The observed resistance patterns are consistent with global and regional analyses, indicating that rising AMR is largely driven by sustained selection pressure from empiric and prolonged use of broad‐spectrum antibiotics, particularly in high‐acuity care settings where stewardship implementation may be variable [12]. In contexts where diagnostic support is limited or delayed, empiric regimens are often continued longer than intended, increasing exposure to high‐priority antibiotics and amplifying selective pressure [13]. This dynamic is especially relevant in tertiary hospitals managing critically ill patients, where clinical urgency may constrain opportunities for early de‐escalation.

Beyond their clinical implications, these findings also reflect a broader biosafety and biosecurity challenge within Indonesian healthcare and other low‐ and middle‐income settings [4–6]. The predominance of *K. pneumoniae* and *A. baumannii* across multiple care settings, coupled with consistently low susceptibility to cephalosporins, fluoroquinolones, and *β*‐lactam/*β*‐lactamase inhibitor combinations, suggests entrenched resistance rather than sporadic emergence.

From a biosecurity perspective, the circulation of MDR, XDR, and potentially pan‐drug‐resistant (PDR) organisms within hospital environments increases the risk of sustained transmission if infection prevention measures are suboptimal [5, 10]. These pathogens pose threats not only to patients but also to healthcare workers and the surrounding community, given their ability to persist on environmental surfaces and to disseminate resistance determinants through horizontal gene transfer [10, 11]. Consequently, rigorous infection control practices, including environmental cleaning, device management, and cohorting, remain essential components of institutional biosecurity alongside antimicrobial stewardship.

### 4.2. Comparisons With National and Regional Surveillance

The resistance patterns observed at YARSI Hospital are broadly consistent with reports from other Indonesian tertiary hospitals, where resistance of *K. pneumoniae* to third‐generation cephalosporins frequently exceeds 50% and carbapenem resistance has shown a rising trajectory [14–16]. Budayanti et al. [14] documented a high burden of MDR *K. pneumoniae* and *P. aeruginosa*, with *A. baumannii* emerging as a dominant ICU pathogen retaining susceptibility largely to amikacin. Subsequent studies have confirmed that *A. baumannii* remains a major challenge in critical care settings, with many isolates exhibiting extensive drug resistance and limited therapeutic options, often restricted to colistin [14, 16, 17]. National surveillance data further indicate a steady increase in carbapenem‐resistant Enterobacterales (CRE), underscoring the expanding scope of resistance beyond individual hospitals [16]. In Surabaya, Utami et al. [18] reported preserved susceptibility of urinary *E. coli* to carbapenems and aminoglycosides, alongside fluoroquinolone resistance exceeding 30%, a pattern concordant with the reduced ciprofloxacin activity against *E. coli* observed in the present study.

These national findings parallel broader Southeast Asian trends, where MDR *K. pneumoniae* predominates among ICU isolates and carbapenem resistance in *A. baumannii* has reached 40%–60% in countries such as Thailand and Vietnam [19, 20]. At the global level, longitudinal surveillance has demonstrated a sustained increase in carbapenem resistance among Enterobacterales over the past decade [21]. Collectively, these comparisons suggest that the resistance profiles observed at YARSI Hospital are not isolated anomalies but reflect shared regional and global pressures, including prolonged exposure to broad‐spectrum antibiotics in critical care environments and limited opportunities for effective de‐escalation.

Importantly, large observational analyses have highlighted a growing reliance on “Watch” and “Reserve” category antibiotics, agents with a higher propensity to select for resistance, particularly in hospital settings managing severe infections [22]. Without robust stewardship oversight, such prescribing patterns may accelerate resistance amplification across institutions. In parallel, contextual reviews have identified gaps in guideline implementation, diagnostic capacity, and policy coordination in LMICs, factors that can further exacerbate resistance trajectories when not addressed systematically [13]. Together, these data indicate that resistance patterns at YARSI Hospital are embedded within broader health‐system dynamics, reinforcing the need for coordinated stewardship, surveillance, and policy responses rather than isolated institutional interventions.

### 4.3. Isolate‐Specific Observations

In contrast to ICU and respiratory isolates, *E. coli* from urine specimens at YARSI Hospital retained relatively high susceptibility to ceftazidime, cefepime, meropenem, and amikacin. This pattern is consistent with other Indonesian and international reports [19, 23, 24] and likely reflects differences in care pathways and cumulative antibiotic exposure between community‐associated urinary tract infections (UTIs) and hospital‐acquired infections. In community‐acquired UTIs, antibiotic courses are generally shorter and narrower in spectrum, which may limit selective pressure compared with prolonged, multiclass exposure typical of critical care settings. These findings suggest that empirical regimens for uncomplicated community‐acquired UTIs remain largely effective. However, the observed reduction in susceptibility to fluoroquinolones and trimethoprim–sulfamethoxazole supports the use of culture‐guided therapy for recurrent, complicated, or healthcare‐associated UTIs, in line with current IDSA recommendations [25, 26].

Evidence from longitudinal antimicrobial stewardship studies indicates that structured stewardship interventions can incrementally improve prescribing appropriateness and reduce selective pressure on Gram‐negative bacilli, including ESBL‐producing organisms, in tertiary care environments [27]. The contrast between susceptibility patterns observed in urinary *E. coli* and those seen in ICU‐associated pathogens in the present study is therefore consistent with the established relationship between antibiotic intensity, duration of exposure, and resistance emergence across different clinical settings.

Among Gram‐positive organisms, *S. epidermidis* and *E. faecalis* demonstrated preserved susceptibility to vancomycin and linezolid, in agreement with regional and global surveillance data [28, 29]. Nevertheless, the frequent recovery of *S. epidermidis* from blood cultures warrants cautious interpretation, as this organism commonly represents contamination rather than true bloodstream infection [30, 31]. Such findings underscore the importance of diagnostic stewardship and strict aseptic blood culture collection practices, both to improve data quality and to avoid unnecessary escalation of antimicrobial therapy driven by misclassified contaminants.

### 4.4. Temporal Trends

Semester‐wise comparison revealed largely stable resistance trends during 2024. However, subtle but epidemiologically relevant changes were observed, including a modest decline in carbapenem susceptibility among ICU‐derived *K. pneumoniae* and increasing resistance of *A. baumannii* to trimethoprim–sulfamethoxazole in the second semester. Similar short‐term temporal fluctuations have been reported in Asian hospital surveillance studies and are thought to reflect cumulative antibiotic selection pressure, shifts in empiric prescribing patterns, and intermittent clonal dissemination within high‐acuity units, rather than abrupt changes in resistance mechanisms [32, 33].

The significant semester‐wise shifts in specimen mix, sample origins, and organism composition, particularly the increased contribution from the emergency department and nonfermenting Gram‐negative bacilli in the second semester, suggest changes in patient flow and case mix, potentially increasing exposure to healthcare‐associated pathogens at earlier stages of admission. Such epidemiological shifts underscore the importance of interpreting antibiogram trends in conjunction with hospital utilization patterns and reinforce the value of routine, stratified antibiogram surveillance to inform empirical therapy and stewardship planning [26, 27].

Broader global analyses indicate that these temporal resistance patterns are shaped by system‐wide determinants, including limited diagnostic capacity, inconsistent enforcement of antimicrobial stewardship policies, and unequal access to effective or last‐line antibiotics. Together, these factors influence antibiotic selection, duration of therapy, and opportunities for de‐escalation, thereby collectively driving resistance dynamics over time, particularly in low‐ and middle‐income healthcare settings [34, 35].

### 4.5. Implications and Limitations

These findings support three critical actions. First, routine, stratified antibiogram reporting is essential to detect emerging resistance patterns and to guide context‐specific empirical therapy, particularly in settings with heterogeneous patient populations and shifting epidemiology [36]. Second, strengthened antimicrobial stewardship programs are needed to curb inappropriate use of broad‐spectrum antibiotics, promote timely de‐escalation, and align prescribing with local resistance data and WHO AWaRe principles [37, 38]. Third, enhanced diagnostic stewardship and strict aseptic sampling practices are required to improve data validity, reduce blood culture contamination, and prevent unnecessary antibiotic escalation driven by misclassified isolates [39, 40].

This study is limited by its single‐center design, retrospective data collection, and the absence of molecular characterization of resistance mechanisms (e.g., carbapenemase genotyping), which constrain causal inference and mechanistic interpretation. Nonetheless, adherence to CLSI M39‐A4 methodology and comprehensive stratification by specimen type and care setting strengthen internal validity and provide actionable institutional insights for stewardship and infection prevention.

By situating YARSI Hospital’s resistance data within Indonesian, Southeast Asian, and global AMR trends, this report offers pragmatic evidence to refine empirical prescribing and stewardship strategies. Collectively, these findings underscore the importance of hospital‐level, stratified antibiogram surveillance as a foundational component of Indonesia’s broader response to AMR [41].

## 5. Conclusion

The 2024 cumulative antibiogram from YARSI Hospital highlights the substantial burden of AMR, predominantly driven by MDR *K. pneumoniae* and *A. baumannii* in critical care settings and invasive infections. These resistance patterns severely restrict empirical treatment options. In contrast, *E. coli* urinary isolates retained higher susceptibility to several key antibiotics, and Gram‐positive organisms remained largely sensitive to glycopeptides and oxazolidinones. Nonetheless, the predominance of resistant Gram‐negatives in the ICU and frequent recovery of *S. epidermidis* from blood cultures underscore persistent vulnerabilities in clinical practice.

These findings strongly support the need for routine, stratified antibiogram reporting and the implementation of locally adapted antimicrobial stewardship programs. Such measures are essential to optimize empirical therapy, improve diagnostic accuracy, and enable continuous surveillance of evolving resistance trends. Strengthening these practices is vital not only for safeguarding patient outcomes at the institutional level but also for contributing to Indonesia’s broader efforts in combating AMR as part of the global health agenda.

## Author Contributions

R.B. contributed to conceptualization, methodology, supervision, validation, and writing (review and editing). M.A.R.P. contributed to conceptualization, data curation, formal analysis, investigation, methodology, visualization, writing (original draft), and writing (review and editing). S.B. contributed to data curation, investigation, resources, and writing (review and editing). N.P. contributed to project administration, resources, supervision, and writing (review and editing). E.Y. contributed to data curation, investigation, resources, and writing (review and editing). N.R.V.B. contributed to data curation, investigation, and writing (review and editing). A.A.S. contributed to data curation, investigation, and writing (review and editing). T.D.F. contributed to data curation, investigation, and writing (review and editing).

## Funding

No funding was received for this manuscript.

## Ethics Statement

This study received ethical approval from the Ethics Committee of YARSI Hospital, Jakarta. The study was conducted in accordance with the ethical standards of the committee and with the principles of the Declaration of Helsinki. Permission to access anonymized laboratory records was obtained from the hospital administration, and all data were deidentified prior to analysis to ensure confidentiality.

## Consent

Given the retrospective design of the study and the exclusive use of deidentified laboratory data, the requirement for individual patient consent was waived by the Ethics Committee.

## Conflicts of Interest

The authors declare no conflicts of interest.

## Data Availability

The data that support the findings of this study are available from the corresponding authors upon reasonable request.
